# Overweight and obesity: prevention in the first 1,000 days of life

**DOI:** 10.1186/1824-7288-41-S2-A44

**Published:** 2015-09-30

**Authors:** Claudio Maffeis

**Affiliations:** 1 Pediatric Diabetes and Metabolic Disorders Unit, Department of Life & Reproduction Sciences, University of Verona, 37126 Verona, Italy

## 

More than 30% of 9-10-year old Italian children are overweight or obese. The American Academy of Pediatrics as well as the Italian Society of Pediatrics proposed some specific targets for both prevention and treatment of childhood obesity. However, consistent evidence suggests that nutrition is the most promising among them. Nutritional intervention may be efficacious at every age but the most sensitive age window is the first 1,000 days of life, i.e., intrauterine period and the first two years after birth. In early life, the delicate phase of anatomic growth and functional development and specialization of the neuroendocrine system that regulates energy balance and body composition may be affected by nutrition. Unfavorable nutrition conditions, lead to a non-physiologic “metabolic programming”, i.e., the maturation of the biochemical pathways regulating metabolism and adiposity, also in a long term perspective. The epigenetic consequences induced by inadequate nutritional exposure makes individuals less resistant to the environmental obesogenic pressure, leading to a morbidity-prone phenotype. What are the nutritional targets in the first 2 years after birth? Prolonged breast feeding and, at weaning, provide complimentary food that are nutritionally adequate and safe. Diet composition should guarantee all macro- and micro-nutrients recommended for optimal growth. In particular, it should be avoided the excess of energy, protein and sugar in respect to requirement as well as the shortage of iron, vitamin D, and lipids, especially long-chain polyunsaturated fatty acids. Moreover, an accurate monitoring of weight and length growth should be always provided, to avoid undesirable acceleration of weight growth velocity in respect to length growth velocity, an important risk factor of obesity. Cow milk should be avoided at least in the first 12 months of life. Parents have a high responsibility in educating they children to correct eating behavior and the role of pediatricians to inform and guide parents in this delicate work is crucial.

